# Genome-Wide Identification and Expression Analysis of the *Shaker* K^+^ Channel Gene Family in Cassava (*Manihot esculenta* Crantz) Under Potassium Stress

**DOI:** 10.3390/plants14142213

**Published:** 2025-07-17

**Authors:** Xianhai Xie, Chenyu Lin, Feilong Yu, Haozheng Li, Jin Xiao, Mingjuan Zheng, Wenquan Wang, Xin Guo

**Affiliations:** School of Tropical Agriculture and Forestry, Hainan University, Haikou 570228, China; xianhaixie0901@163.com (X.X.); lcy1179456412@163.com (C.L.); yu1484754611@163.com (F.Y.); lihaozheng0212@163.com (H.L.); 18804055876@163.com (J.X.); zm-72580@outlook.com (M.Z.)

**Keywords:** *Shaker* K^+^ channel gene, cassava, potassium stress, expression patterns, potassium utilization

## Abstract

Shaker K^+^ channel proteins are responsible for potassium (K^+^) uptake and transport, playing a critical role in plant growth, development, and adaptation to K^+^ deficiency. Cassava, a key tropical root crop, is known for its characteristic of resilience to nutrient-poor soil and drought stress. However, the Shaker K^+^ channel gene family in cassava has not yet been characterized. In this study, 13 Shaker channel genes were identified from the near telomere-to-telomere (T2T) cassava genome using bioinformatics analysis. Phylogenetic relationships classified these genes into five distinct subfamilies, and all encoded proteins contained the conserved GYGD/GYGE motif typical of Shaker channels. Protein interaction network predictions revealed potential interactions among the Shaker family, as well as with the potassium transporter HAK5. Tissue-specific expression pattern analysis showed that *MeGORK* and *MeAKT1.2* were expressed in all tissues. Furthermore, quantitative real-time PCR (qRT-PCR) analysis was conducted to examine the transcriptional levels of *Shaker* K^+^ channel gene family members in the roots and leaves of two cassava germplasms with different low-potassium tolerance after one month of low-potassium treatment. The results revealed that *MeAKT1.2*, *MeAKT2.2*, and *MeKAT1* exhibited distinct expression patterns between the two germplasms, with higher expression levels observed in the potassium-tolerant germplasm. Therefore, these three genes may serve as important candidate genes for potassium stress tolerance in cassava. In summary, this study provides valuable insights into the characteristics and biological functions of the *Shaker* K^+^ channel gene family in cassava and identifies potential candidate genes for breeding or engineering potassium-efficient cassava cultivars.

## 1. Introduction

Potassium (K^+^) is a crucial macronutrient that significantly improves crop yield and quality across various plant species. In root crop like cassava, potassium fertilization enhances the translocation of assimilates and starch biosynthesis, resulting in increased total biomass, storage root yield, and harvest index [1,2,3]. Potassium application can increase the number of storage roots and the weight of individual storage roots in sweet potato [4]. Starch plays a central role in root crops such as cassava, sweet potato, and potato, serving as the primary energy storage form and significantly influencing the economic value and food processing properties of these crops. Starch synthesis mainly occurs through the starch–sucrose metabolic pathway [5]. Potassium application enhances the activity and transcription levels of sucrose synthase (SuSy) and adenosine-diphosphate-glucose pyrophosphorylase (AGPase), thereby promoting starch synthesis in sweet potato [6]. For instance, potassium application has been shown to raise rice yields by 4.2–8.9% and rapeseed yields by 7.5–32.6% [7]. In potato, potassium not only boosts the starch content in tubers and shoot biomass but also improves nutrient uptake and overall yield [8]. Beyond promoting growth and productivity, potassium also contributes to plant responses against various biotic and abiotic stresses such as drought, cold, and salinity [9,10]. As the most abundant cation in the plant cytoplasm, potassium plays a vital role in maintaining cell osmotic pressure, establishing osmotic gradients in phloem vascular systems, sustaining root pressure in xylem vascular systems, and regulating stomatal aperture [11]. Due to the lack of a sodium-potassium exchange system in plants, specialized potassium (K^+^) channels have evolved to facilitate K^+^ uptake and efflux, maintaining intracellular ion homeostasis and supporting plant development. Among these channels, the *Shaker*-type K^+^ channels represent a major class of voltage-gated K^+^ channels involved in these essential processes.

The *Shaker* K^+^ channel gene family were first discovered in fruit flies [12]. Their characteristic structure underpins their selective K^+^ transport function: four α-subunits form a tetrameric channel with a central pore. Each subunit comprises an N-terminal region, a C-terminal region, six transmembrane domains (S1–S6), and a conserved pore-forming region of approximately 60 amino acids [13,14]. The S5 and S6 segments, along with the intervening pore (P) domain, constitute the channel’s ion-selective filter. The S4 segment, rich in positively charged residues, acts as a voltage sensor that regulates channel gating by undergoing conformational changes in response to membrane potential [14]. *AtAKT1* is the first cloned potassium channel gene of the *Shaker* family, located on chromosome 2 of Arabidopsis thaliana. It contains 10 introns and 11 exons, encoding a protein with 857 amino acid residues [15,16]. In rice, *OsAKT1* is primarily expressed in roots and leaves [17], where it plays a key role in potassium uptake.

Potassium uptake and translocation are particularly crucial under drought conditions, as they help mitigate transpirational water loss and maintain cellular homeostasis. However, due to potassium fixation by soil silicates, K^+^ is often present at low levels in agricultural soils. Therefore, identifying K^+^ transport-related genes in cassava, particularly Shaker K^+^ channels, is of practical importance in improving potassium use efficiency and stabilizing starch yield under low-K^+^ conditions. While the Shaker gene family has been characterized in several plant species such as *Arabidopsis thaliana* [18], *Oryza sativa* [19], *Setaria italica* [20], *Glycine max* [21], and mango [22], no comprehensive study has been conducted yet in cassava. Cassava (*Manihot esculenta* Crantz) is a vital tropical and subtropical crop widely cultivated in African countries. Its starchy storage roots serve as a staple food for over one billion people worldwide [23]. Cassava is characterized by high photosynthetic efficiency, strong adaptability to nutrient-poor soil and drought stress, and its ability to grow on marginal lands. With yields often surpassing those of cereals, cassava is considered a promising crop in mitigating the effects of climate change and ensuring future global food security. In this study, we performed a genome-wide identification of *Shaker* K^+^ channel genes in cassava using the near T2T (v8.1) genome. In total, 13 members were identified and further characterized through phylogenetic, conserved motif, promoter, and protein interaction network analyses. In addition, their transcriptional responses to one-month low-potassium treatment were examined using quantitative real-time PCR (qRT-PCR). This work lays a theoretical foundation for understanding the role of K^+^ channels in cassava and provides candidate genes for the development of potassium-efficient cultivars.

## 2. Results

### 2.1. Genome-Wide Identification of Shaker K^+^ Channel Gene Family Members in Cassava

**Figure 1:**
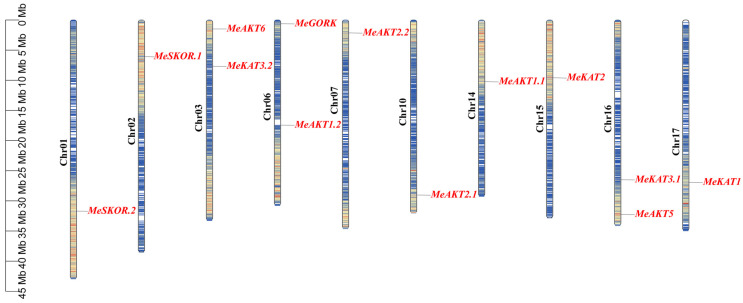
Chromosomal distribution of 13 *MeShaker* K^+^ channel gene family members across 10 cassava chromosomes. Thirteen identified *MeShaker* K^+^ channel genes were mapped to the cassava genome and visualized on 10 chromosomes. Each gene’s physical position was determined based on the latest genome annotation data. The scale bar on the left indicates chromosome length in megabases (Mb), providing a reference for gene localization. A color gradient overlay along each chromosome represents gene density, with darker shades indicating higher gene-rich regions. The figure illustrates the non-random distribution pattern of *MeShaker* genes, suggesting potential chromosomal hotspots for ion transport regulation in cassava.

Using homology-based alignment and genome-wide scanning with Shaker-specific domains, we identified 13 *Shaker* K^+^ channel genes from the near T2T reference genome (v8.1) of cassava. These genes encode proteins containing several conserved domains, including the Ion_trans_2 domain, the cyclic nucleotide-binding (cNMP) domain, and the ankyrin (ANK) repeat domain—except for *MeKAT1*, *MeKAT3.1*, and *MeKAT3.2*, which lack the ANK domain—as well as the typical KHA structure. The 13 *Shaker* K^+^ channel genes are unevenly distributed across 10 of the 18 cassava chromosomes, with two genes each mapped to chromosomes 3 and 16, and one gene each located on chromosomes 1, 2, 6, 10, 14, 15, and 17. Standardized gene names were assigned to cassava Shaker homologs based on sequence homology with *Arabidopsis* counterparts (Table 1; Figure 1). Subcellular localization analysis predicted that the majority of cassava Shaker proteins were targeted to the chloroplasts and plasma membrane. Transmembrane domain analysis indicated that these proteins contain either four or six predicted transmembrane helices (Table 1). Furthermore, tertiary structure modeling revealed considerable variation in overall folding patterns and domain architectures among the family members (Figure S1), suggesting potential functional divergence. The encoded proteins also exhibit notable differences in size: MeAKT5 is the largest, comprising 902 amino acids with a predicted molecular weight of 101.91 kDa, while MeAKT3.2 is the smallest, consisting of 630 amino acids. The theoretical isoelectric points (pI) of these proteins range from 5.98 to 8.47 (Table 1), implying that they may function in distinct subcellular microenvironments.

### 2.2. Phylogenetic Analysis of the Shaker K^+^ Channel Genes

**Figure 2:**
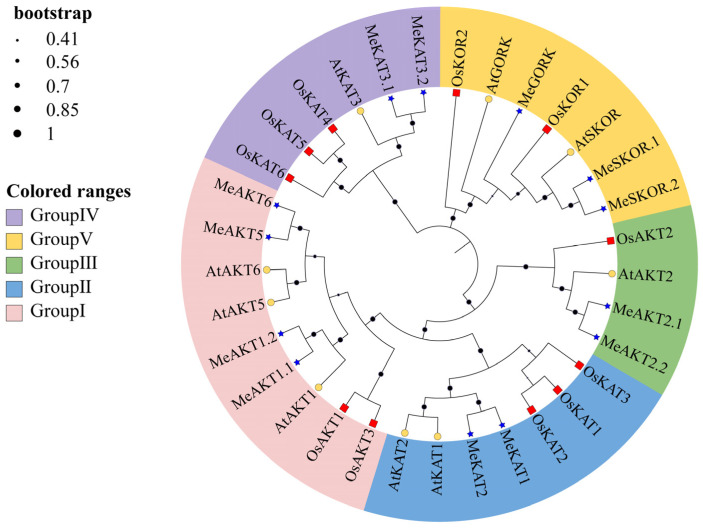
Phylogenetic tree of Shaker K^+^ channel proteins from *Oryza sativa*, *Arabidopsis thaliana*, and *Manihot esculenta*, constructed using the maximum likelihood (ML) method with 1000 bootstrap replicates. Red squares represent *Oryza sativa* Shaker K^+^ channel proteins, yellow circles denote *Arabidopsis thaliana* proteins, and blue pentagons indicate cassava proteins. The tree shows the classification of Shaker proteins into five distinct subgroups (Group I–Group V), with Group I shown in pink, Group II in blue, Group III in green, Group IV in purple, and Group V in yellow.

To investigate the evolutionary relationships of Shaker K^+^ channel proteins in cassava, a phylogenetic analysis was performed using MEGA12 software. Full-length amino acid sequences of Shaker proteins from cassava, *Arabidopsis thaliana*, and *Oryza sativa* were aligned, and a maximum likelihood phylogenetic tree was constructed (Figure 2). Multiple sequence alignment revealed that all 13 cassava Shaker proteins possess the highly conserved GYGD/GYGE motif, a hallmark of this gene family (Figure S2). Phylogenetic classification divided the cassava Shaker proteins into five distinct subfamilies. Group I contains four members (MeAKT6, MeAKT1.2, MeAKT1.1, and MeAKT5); Group II includes two members (MeKAT2 and MeKAT1); Group III comprises two members (MeAKT2.2 and MeAKT2.1); Group IV consists of three members (MeSKOR.2, MeSKOR.1, and MeGORK); and Group V includes two members (MeKAT3.2 and MeKAT3.1). This phylogenetic grouping is consistent with previous studies in other plant species, indicating that the Shaker gene family is highly conserved during evolution and that the cassava Shaker proteins retain similar structural and functional characteristics.

### 2.3. Homology Analysis of Shaker K^+^ Channel Genes

**Figure 3:**
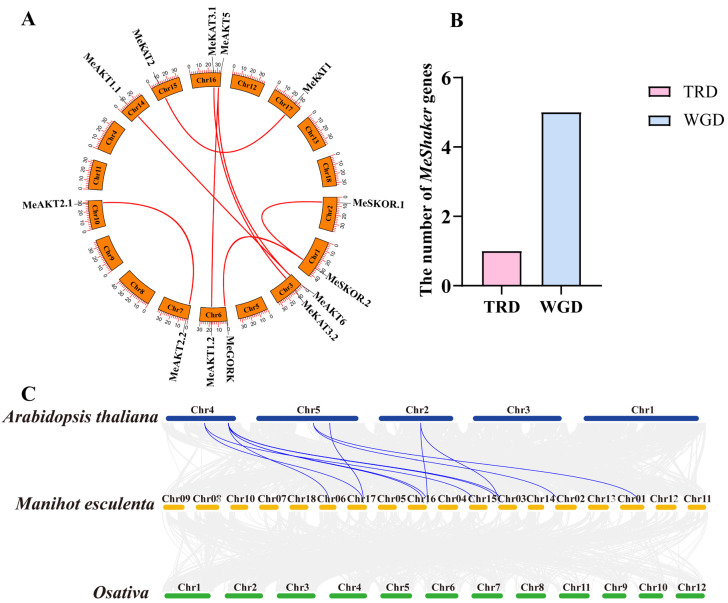
(**A**) Segmental duplication analysis of *Shaker* K^+^ channel genes in cassava; red curves represent pairs of segmentally duplicated genes. (**B**) Classification and quantification of gene duplication events among cassava *Shaker* K^+^ channel gene family members. (**C**) Synteny analysis of *Shaker* K^+^ channel genes in *Arabidopsis thaliana*, *Manihot esculenta*, and *Oryza sativa*. Gray lines in the background indicate syntenic blocks between cassava and the other plant genomes, while red lines highlight syntenic gene pairs between *Manihot esculenta* and *Arabidopsis thaliana*.

The 13 cassava *Shaker* K^+^ channel genes were mapped to eight duplicated regions distributed across linkage groups 1, 2, 3, 6, 7, 10, 14, 15, 16, and 17. Within these regions, several gene pairs were identified, including *MeSKOR.2/MeSKOR.1*, *MeSKOR.2/MeGORK*, *MeAKT6/MeAKT1.1*, *MeAKT6/MeAKT5*, *MeKAT3.2/MeKAT3.1*, *MeAKT1.2/MeAKT5*, *MeAKT2.2/MeAKT2.1*, and *MeKAT2/MeKAT1* (Figure 3A). To further investigate the origin of gene duplication, gene duplication modes were classified using the DupGen_finder tool. The analysis revealed that six gene pairs were derived from whole-genome duplication (WGD) events (e.g., *MeShaker1/2*, *MeShaker3/6*), while one gene pair resulted from tandem duplication (TRD) (*MeShaker6/9*) (Figure 3B). To examine the evolutionary constraints acting on these duplicated genes, nonsynonymous (Ka) and synonymous (Ks) substitution rates were calculated, and Ka/Ks ratios were determined for each duplicated pair (Table S1). The Ka/Ks ratios ranged from 0.17 to 0.39, all below 1.0, indicating that these genes had undergone strong purifying (negative) selection during evolution. These results suggest that gene duplication—particularly through WGD—has played a major role in the expansion and evolutionary conservation of the Shaker gene family in cassava.

To further explore the evolutionary trajectory of the *Shaker* gene family in cassava, a comparative synteny analysis was performed using representative dicot (*Arabidopsis thaliana*) and monocot (*Oryza sativa*) species. The results showed that 11 of the 13 identified *MeShaker* genes exhibited collinear relationships with homologous genes in *Arabidopsis*, whereas no syntenic relationships were detected with *Oryza sativa* (Figure 3C). This pattern suggests that the *Shaker* gene family in cassava likely originated or underwent major expansion after the divergence between dicotyledonous and monocotyledonous lineages. These findings provide insights into the evolutionary conservation of the *Shaker* gene family within dicots and highlight lineage-specific retention or expansion events in cassava.

### 2.4. Structural Features and Conserved Motif Analysis of Cassava Shaker K^+^ Channel Genes

To elucidate the structural characteristics of the *Shaker* K^+^ channel gene family in cassava, exon–intron structures were analyzed using TBtools-II. The genomic lengths of the cassava Shaker genes range from 5.5 kb to 16.5 kb, and all genes contain complete 5′ and 3′ untranslated regions (UTRs). Exon–intron structure analysis revealed subfamily-specific patterns: Group IV and Group V members each possess 13 exons; Group I members contain 11–12 exons; Group II has 10–11 exons; and Group III genes uniformly contain 11 exons. These patterns suggest that exon–intron structures are relatively conserved within subfamilies, potentially reflecting shared evolutionary origins and functional constraints.

**Figure 4:**
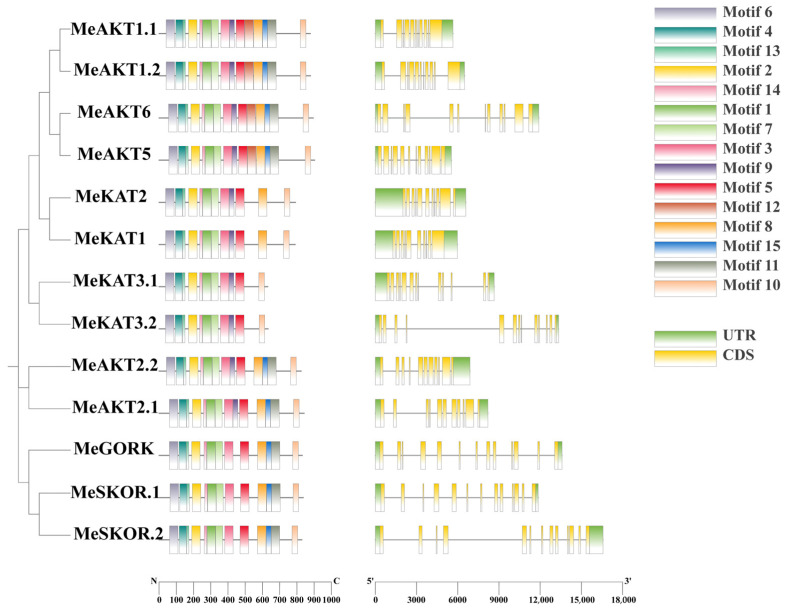
Conserved motif composition and gene structure analysis of 13 cassava Shaker K^+^ channel genes. A maximum likelihood phylogenetic tree (**left**) based on full-length protein sequences. To the right of each protein name, colored boxes indicate the positions of 15 conserved motifs identified by MEME, with each motif assigned a unique color reflecting its order and relative location within the sequence. The adjacent gene structure panel displays exon–intron organization: yellow boxes denote coding sequences (CDS), green boxes indicate untranslated regions (UTRs), and black lines represent introns. The scale bar beneath each panel shows the protein length in amino acids (**left**) and gene length in base pairs (**right**). The phylogenetic tree on the left was constructed using the maximum likelihood (ML) method with 1000 bootstrap replicates.

Motif composition analysis was performed using the MEME suite, revealing that members within the same subfamily generally shared similar motif patterns, indicative of conserved functional domains. All cassava Shaker proteins were found to contain motifs 1–7, 10, 13, and 14, indicating that these 10 motifs are evolutionarily conserved and may be essential to the structural and functional integrity of Shaker K^+^ channels in cassava. Notably, motif 12 was specifically present in Group I members, whereas motifs 11 and 15 were absent from Group II, and Group III lacked motifs 8, 11, and 15, further supporting structural divergence among subfamilies (Figure 4). These variations in motif composition may underlie functional specialization among different Shaker channel subtypes.

### 2.5. Promoter Analysis of Cassava Shaker K^+^ Channel Genes

To investigate the potential regulatory mechanisms governing the expression of cassava *Shaker* K^+^ channel genes, cis-acting elements within their promoter regions were analyzed using the PlantCARE database. A total of 45 distinct cis-elements were identified across the 13 *Shaker* K^+^ channel gene promoters and classified into four major functional categories: light-responsive elements, hormone-responsive elements, growth and development-related elements, and stress-responsive elements.

**Figure 5:**
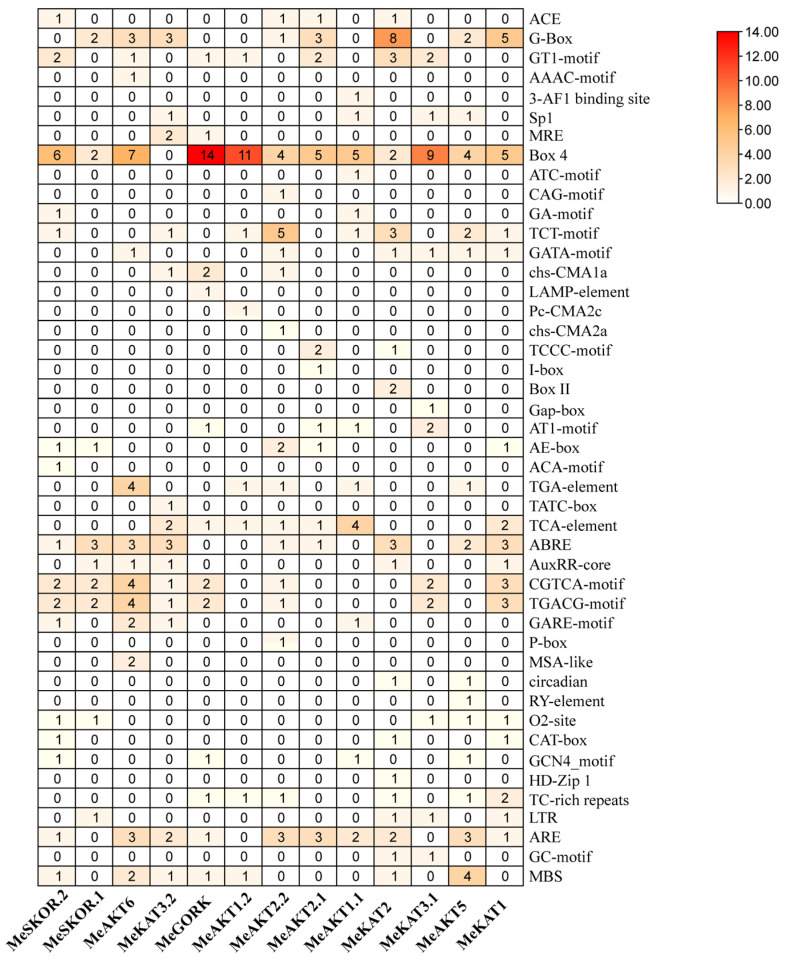
Prediction of cis-acting elements in the promoters of cassava *Shaker* K^+^ channel genes. Columns represent individual *Shaker* K^+^ channel genes, while rows correspond to distinct cis-regulatory elements identified from their promoter regions. The color intensity indicates the occurrence frequency of each element type, with darker colors representing a higher frequency and lighter colors indicating a lower frequency.

Light-responsive elements were the most abundant, encompassing 22 types, including G-Box, Box 4, and TCT-motif, indicating that light signaling may play a key regulatory role in the expression of cassava *Shaker* K^+^ channel genes. Hormone-responsive elements formed the second-largest group, comprising nine types, such as abscisic acid-responsive elements (ABRE), methyl jasmonate-responsive elements (CGTCA-motif), and jasmonic acid-responsive elements (TGACG-motif). Growth and development-related elements comprised seven types, notably MSA-like, O2-site, and CAT-box, which are often associated with cell cycle regulation and tissue-specific expression. Stress-responsive elements were the least abundant, with only five types identified, including drought- and salt-stress-responsive TC-rich repeats, low-temperature-responsive LTR elements, and drought-inducible MBS elements (Figure 5).

Collectively, these results indicate that cassava *Shaker* K^+^ channel genes are potentially regulated by a complex network of environmental and endogenous signals, including light, phytohormones, developmental cues, and abiotic stress stimuli. This underscores their likely functional relevance in plant growth, physiological regulation, and environmental adaptation.

### 2.6. Interaction Network of Cassava Shaker K^+^ Channel Proteins

**Figure 6:**
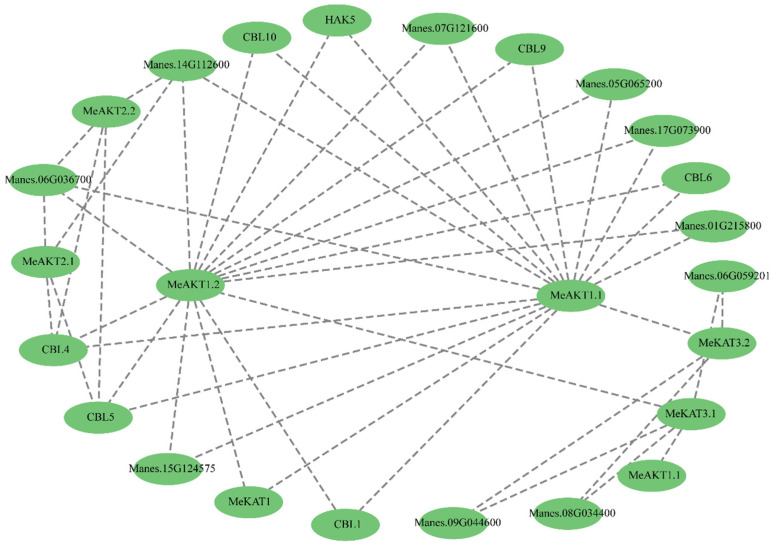
Protein–protein interaction (PPI) network analysis of cassava Shaker K^+^ channel proteins. The PPI network was constructed using predicted interactions among cassava Shaker K^+^ channel proteins based on STRING database analysis. Each node represents a protein, and edges between nodes indicate predicted or known interaction relationships. The network illustrates both direct and indirect associations, reflecting potential functional cooperation among members of the MeShaker family. Nodes represent proteins involved in direct interactions, while edges indicate interaction relationships.

PPI networks of the cassava Shaker K^+^ channel gene family were predicted using the PPI analysis module in TBtools-II and subsequently visualized with Cytoscape software. As shown in Figure 6, MeAKT1.2 and MeAKT1.1 occupy central positions within the interaction network. These two proteins were predicted to interact with calcineurin B-like proteins (CBL1 and CBL9) and the high-affinity potassium transporter HAK5, among others. Their central positioning and extensive predicted interactions suggest that MeAKT1.2 and MeAKT1.1 may serve key roles in potassium signaling and ion homeostasis in cassava.

Additionally, MeAKT2.2 and MeAKT2.1 were specifically predicted to interact specifically with CBL4 and CBL5, respectively, implying potential participation in distinct calcium-mediated signaling pathways (Figure 6). These interaction patterns reveal potential functional diversification among cassava Shaker family members and suggest that different Shaker proteins may integrate specific signaling cues to regulate K^+^ transport and cellular responses.

In summary, these PPI predictions provide insights into the complex regulatory landscape in cassava Shaker proteins and underscore their possible involvement in both shared and specialized physiological processes.

### 2.7. Tissue-Specific Expression Analysis of the Cassava Shaker K^+^ Channel Genes

**Figure 7:**
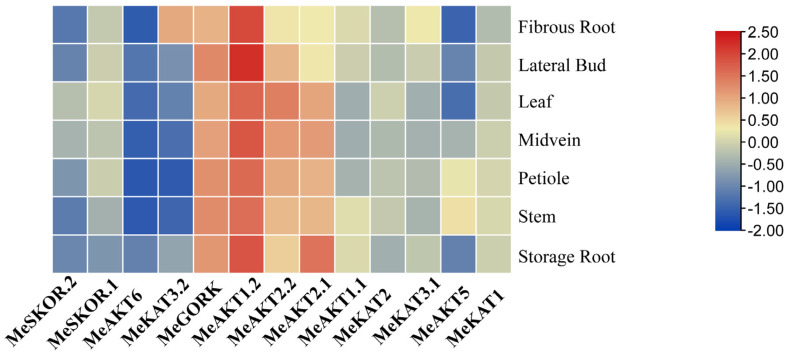
Expression pattern analysis of cassava *Shaker* K^+^ channel genes across different tissues. The expression profiles of *MeShaker* K^+^ channel genes were analyzed across various cassava tissues, including leaves, stems, roots, storage roots, and shoot apices, using publicly available RNA-seq datasets. Gene expression levels were quantified as TPM, followed by log_2_ transformation to normalize the data and enhance the visualization clarity. The resulting heatmap displays the relative expression levels of each gene across tissues, where red indicates high expression and blue indicates low expression.

To investigate the tissue-specific expression patterns of the cassava *Shaker* K^+^ channel gene family, publicly available RNA-seq datasets were analyzed, and a heatmap was generated to visualize transcript abundance across various tissues. The results revealed that *MeAKT1.2* exhibited consistently high expression levels in all examined tissues, suggesting a broad role in developmental processes and physiological regulation throughout the plant. *MeGORK* was also ubiquitously expressed, with particularly elevated transcript levels in stem tissues, implying a potential function in stem-specific potassium homeostasis (Figure 7).

In contrast, *MeAKT2.2* was expressed across all tissues, with the highest levels detected in leaves, whereas *MeAKT2.1* displayed predominant expression in storage roots, indicating functional specialization among these paralogs. *MeKAT3.2* displayed exclusive expression in fibrous roots, suggesting a root-specific function. *MeAKT5* was mainly expressed in petioles and stems, with lower expression in other tissues. In contrast, *MeSKOR.2*, *MeSKOR.1*, *MeAKT6*, *MeAKT1.1*, *MeKAT2*, *MeKAT3.1*, and *MeKAT1* exhibited no detectable expression in the tissues analyzed, indicating either tissue-specific activation under specific conditions or functional redundancy (Figure 7).

These findings underscore the diverse and tissue-specific expression profiles of cassava *Shaker* K^+^ channel genes, suggesting functional specialization in potassium transport and signaling across different organs.

### 2.8. Phenotypic Changes in Cassava Under Potassium Stress

**Figure 8:**
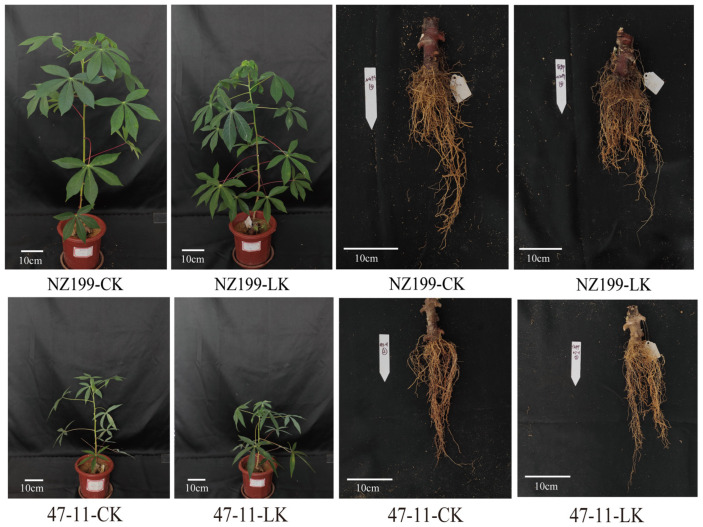
Phenotypic variations in NZ199 (low-potassium-tolerant germplasm) and 47-11 (potassium-sensitive germplasm) under different potassium treatment conditions. Representative phenotypes of two cassava germplasms, NZ199 (tolerant to low-potassium stress) and 47-11 (sensitive to potassium deficiency), were recorded under two potassium treatment conditions: control (CK, 10 mM K^+^) and low potassium (LK, 0.01 mM K^+^). Plants were grown under identical conditions, and phenotypic differences were documented at the same developmental stage.

**Figure 9:**
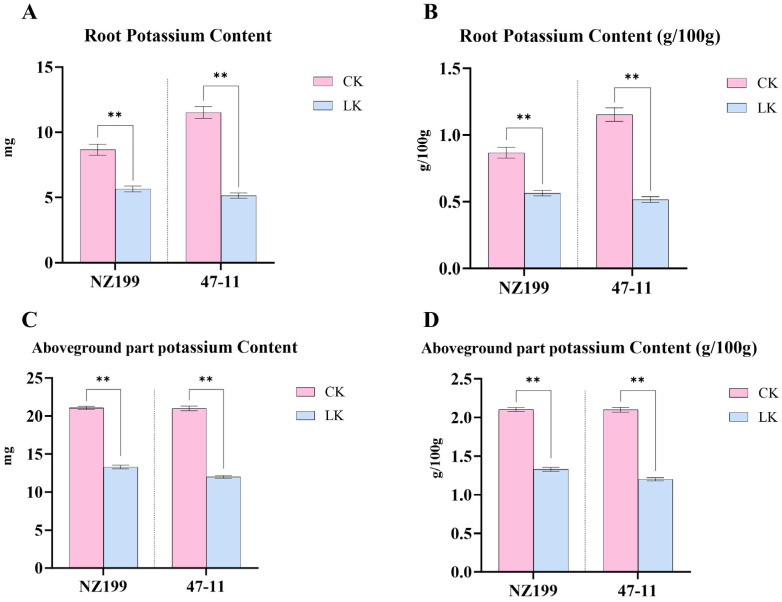
Potassium accumulation and content in the roots and shoots of low-potassium-tolerant germplasm NZ199 and low-potassium-sensitive germplasm 47-11 under potassium treatments. (**A**) Root potassium accumulation; (**B**) root potassium content; (**C**) shoot potassium accumulation; (**D**) shoot potassium content. (CK, potassium concentration at 10 mM; LK, potassium concentration at 0.01 mM). Error bars represent the standard deviation of three biological replicates. Statistical significance was determined by one-way ANOVA, where * denotes *p* < 0.05, ** denotes *p* < 0.01.

To assess the physiological responses of cassava to potassium deficiency, two germplasms with contrasting potassium tolerance, NZ199 (tolerant) and 47-11 (sensitive), were subjected to potassium stress treatments. Phenotypic observations revealed that both germplasms experienced a reduction in plant height and an increase in fibrous root development under low-potassium conditions. Notably, the potassium-sensitive germplasm 47-11 exhibited a more pronounced decline in plant height compared to NZ199 (Figure 8 and Figure S3). Quantitative measurements of potassium content and accumulation in shoot tissues under both control and low-potassium treatments demonstrated that potassium stress significantly reduced shoot potassium content and accumulation in both germplasms (Figure 9C,D). Furthermore, when compared to the tolerant NZ199, the sensitive germplasm 47-11 displayed a significantly greater reduction in shoot potassium content and root potassium accumulation, indicating a compromised ability to maintain potassium homeostasis under stress conditions. These findings highlight genotype-dependent differences in potassium uptake and distribution in cassava, which may underlie variations in potassium stress tolerance (Figure 9A,B).

### 2.9. Expression Patterns of Cassava Shaker Genes Under Potassium Stress

**Figure 10:**
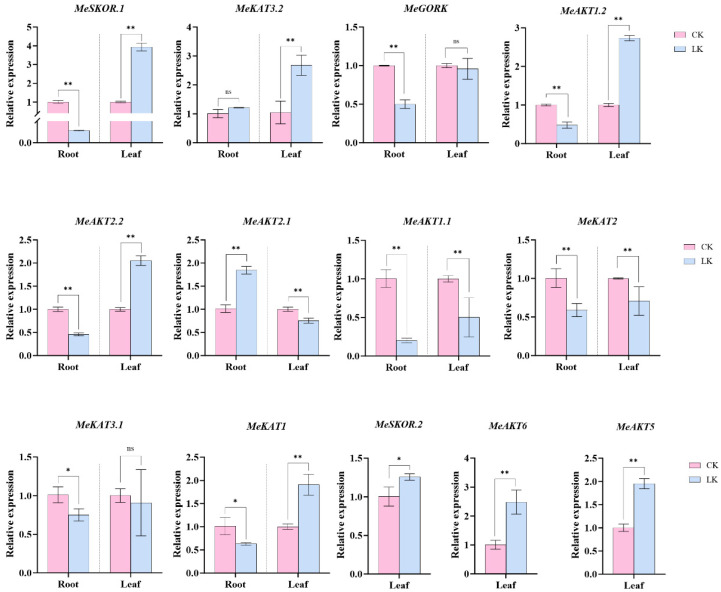
Expression analysis of cassava *Shaker* K^+^ channel gene family members. Expression analysis of genes detected in both roots and leaves of NZ199 (low-potassium-tolerant germplasm) under potassium treatment (CK, potassium concentration at 10 mM; LK, potassium concentration at 0.01 mM), with *MeActin* as the reference gene [24]. The error bars represent the average variance of three biological replicates. Statistical significance was determined by one-way ANOVA, where * denotes *p* < 0.05, ** denotes *p* < 0.01, and ns indicates no significant difference.

To investigate the transcriptional level of cassava *shaker* K^+^ channel genes to potassium deficiency, qRT-PCR analysis was performed on 13 *Shaker* genes in two cassava germplasms (NZ199 and 47-11) subjected to prolonged low-potassium stress. In the potassium-tolerant germplasm NZ199, all but three genes (*MeSKOR.2*, *MeAKT6*, and *MeAKT5*) were responsive in both roots and leaves. Specifically, *MeSKOR.1*, *MeAKT1.2*, *MeAKT2.2*, and *MeKAT1* were significantly downregulated in roots but upregulated in leaves. In contrast, *MeGORK*, *MeAKT1.1*, *MeKAT2*, and *MeKAT3.1* were downregulated in both roots and leaves, despite being induced at the transcriptional level under potassium deficiency. *MeAKT2.1* showed a divergent response, with marked upregulation in roots and downregulation in leaves (Figure 10).

**Figure 11:**
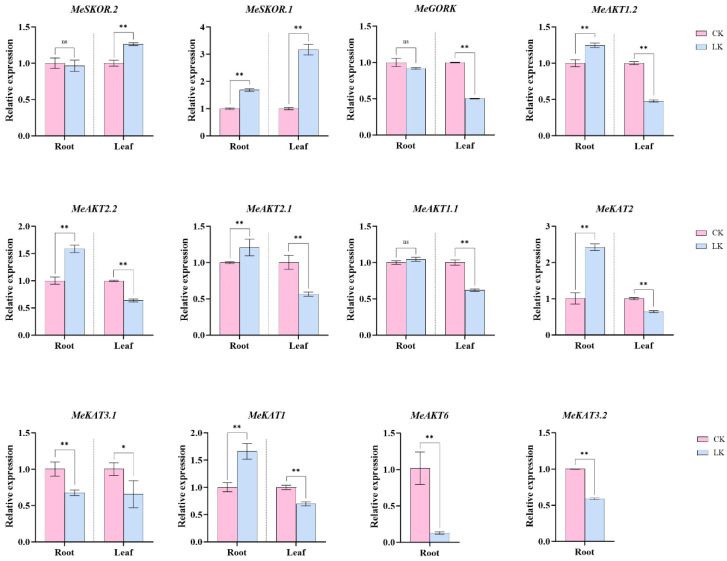
Expression analysis of cassava *Shaker* K^+^ channel gene family members. Expression analysis of genes detected in both roots and leaves of 47-11 (potassium-sensitive germplasm) under potassium treatment (CK, potassium concentration at 10 mM; LK, potassium concentration at 0.01 mM), with *MeActing* as the reference gene [24]. The error bars represent the average variance of three biological replicates. Statistical significance was determined by one-way ANOVA, where * denotes *p* < 0.05, ** denotes *p* < 0.01, and ns indicates no significant difference.

In the potassium-sensitive germplasm 47-11, *MeAKT5* transcripts were undetectable in both roots and leaves, while *MeAKT6* and *MeKAT3.2* were specifically induced in roots only. The remaining 10 genes were differentially expressed in both tissues. Among them, *MeAKT1.2*, *MeAKT2.2*, *MeAKT2.1*, and *MeKAT1* were significantly downregulated in leaves but markedly upregulated in roots. *MeSKOR.1* and *MeSKOR.2* were upregulated in leaves, whereas *MeGORK* and *MeKAT3.1* were downregulated in both tissues (Figure 11). Interestingly, *MeAKT1.2*, *MeAKT2.2*, and *MeKAT1* exhibited opposite expression patterns between the two germplasms, which may contribute to their differing potassium tolerance.

## 3. Discussion

Members of the *Shaker* K^+^ channel gene family have been identified in various plant species, including *Arabidopsis*, *Setaria italica*, *Oryza sativa*, and *Glycine max*. As a major tropical root crop, cassava depends heavily on potassium for storage root development. *Shaker* genes encode K^+^ channel proteins that play crucial roles in potassium uptake, transport, and stress response. Given that the expansion of storage roots is vital for cassava yield, and potassium tolerance facilitates this process, understanding the molecular basis of K^+^ transport is of high agronomic importance. However, comprehensive studies on the Shaker gene family in cassava remain limited. In this study, we systematically identified 13 *MeShaker* K^+^ channel genes from the near-complete T2T cassava genome and performed in-depth analyses of their phylogenetic relationships, gene structures, collinearity, promoter cis-elements, protein interaction networks, and tissue-specific expression profiles.

### 3.1. Evolutionary Conservation of the Shaker K^+^ Channel Gene Family in Plants

Phylogenetic analysis classified cassava Shaker proteins into five subfamilies, consistent with classifications in *Arabidopsis*, *Oryza sativa*, and *Setaria italica* (Figure 2), suggesting that the Shaker gene family is evolutionarily conserved across species. Synteny analysis further revealed a higher degree of collinearity between cassava and *Arabidopsis* Shaker genes than with *Oryza sativa*, reflecting closer evolutionary relationships among dicot species. Structural analyses showed that cassava *Shaker* genes possess well-conserved exon–intron structures and motif compositions (Figure 4), which are consistent with findings in other species [20,21]. These results collectively support both evolutionary and functional conservation within the Shaker gene family.

### 3.2. Functional Implications in Stress Resistance

Cis-acting elements in promoter regions are crucial regulators of gene expression and play essential roles in modulating growth, development, and stress responses [25]. Promoter analysis revealed the presence of several ABA-responsive (ABRE) elements in most *MeShaker* K^+^ channel genes, although these were absent in *MeGORK*, *MeAKT1.1*, *MeAKT1.2*, and *MeKAT3.1*. Additionally, several abiotic stress-related cis-elements, such as TC-rich repeats, LTRs (low-temperature responses), and MBS (MYB binding sites related to drought) (Figure 5), were also identified. These findings suggest that *MeShaker* K^+^ channel genes may participate in K^+^ uptake and transport during abiotic stress adaptation.

HAK5, a member of the KUP/HAK/KT potassium transporter family, plays a central role in high-affinity K^+^ uptake under potassium-deficient conditions [21,26]. Under K^+^-sufficient conditions, HAK5 expression is repressed, whereas it is rapidly induced under K^+^-deficient conditions [27]. Previous studies in *Arabidopsis* and poplar have shown that the CBL1/9–CIPK6/16/23 signaling module activates AKT1 to enhance K^+^ uptake under low-potassium conditions [28,29]. In vivo experiments further confirmed that HAK5 is regulated by the CBL1/9–CIPK module [30]. In the present study, protein–protein interaction network predictions revealed that MeAKT1.1 and MeAKT1.2 interact with both HAK5 and CBL1/9, suggesting that they may form a functional complex within the CBL–CIPK–Shaker module to regulate K^+^ uptake under potassium deficiency and abiotic stress (Figure 6).

In addition to uptake, Shaker proteins are also involved in tissue-specific K^+^ redistribution under stress. For instance, in rice, *OsAKT2* contributes to the movement of K^+^ from stems to roots under salt stress, helping to maintain K^+^/Na^+^ homeostasis [19,31]. In *Arabidopsis*, *AtAKT1* mediates root epidermal K^+^ uptake, while *AtSKOR* mediates xylem loading for long-distance transport to shoots under stress [32,33]. In cassava, heatmap analysis of publicly available RNA-seq data indicated that *MeGORK* and *MeAKT1.2* are expressed in all tissues, with *MeAKT1.2* showing the highest expression, implying a key role in whole-plant K^+^ transport (Figure 7).

Moreover, under low-potassium stress, the low-potassium-tolerant germplasm exhibited differential gene expression patterns, with *MeSKOR.1*, *MeAKT1.2*, *MeAKT2.2*, *MeAKT2.1*, and *MeKAT1* showing low expression in roots but high expression in leaves. In contrast, the expression levels of *MeAKT1.2*, *MeAKT2.2*, and *MeKAT1* were consistently low in the leaves of potassium-sensitive germplasm (Figure 10 and Figure 11). These findings suggest that these genes may be involved in the redistribution of K^+^ during the plant’s response to abiotic stress, which could explain the observed differences in potassium accumulation between the aerial parts and root systems of the two germplasms. This mechanism may be one of the factors contributing to their differing tolerance to potassium stress.

## 4. Materials and Methods

### 4.1. Identification, Physicochemical Properties, and Chromosomal Localization of Cassava Shaker K^+^ Channel Gene Family Members

The genome and annotation files of cassava and *Arabidopsis* were downloaded from the JGI website (https://phytozome-next.jgi.doe.gov/ accessed on 13 June 2024) [34], while the protein sequences of *Arabidopsis* Shaker gene family members were obtained from the TAIR website (https://www.arabidopsis.org/ accessed on 13 June 2024). These sequences were submitted to InterPro for domain identification, including the Ion_trans_2 domain (PF00520), cNMP domain (accession PF00027), ANK domain (accession PF12796), and KHA domain (accession PF11834). Using TBtools-II software [35], the protein sequences of *Arabidopsis* Shaker gene family members were used for local BLAST searches with a threshold of 1 × 10^−5^, yielding candidate protein sequences. The initially obtained protein sequences were uploaded to the NCBI database for further alignment and refinement. The final candidate protein sequences were then submitted to NCBI-CDD (https://www.ncbi.nlm.nih.gov/Structure/bwrpsb/bwrpsb.cgi accessed on 16 June 2024) to confirm the presence of complete domains. Additionally, the HMMER program embedded in TBtools-II was employed to identify cassava Shaker proteins, with a threshold set to 1 × 10^−5^, ensuring the comprehensive and accurate identification of the cassava Shaker gene family members. Protein mass and isoelectric points were predicted using the ExPASy Compute pI/Mw tool [36] available on the ExPASy website (https://web.expasy.org/protparam/ accessed on 16 June 2024). The subcellular localization of MeShaker proteins was predicted using the WOLF PSORT tool [37] (https://wolfpsort.hgc.jp/ accessed on 16 June 2024), and transmembrane domain predictions were performed using TMHMM [38] (https://services.healthtech.dtu.dk/services/TMHMM-2.0/ accessed on 16 June 2024). Chromosomal localization was visualized using TBtools-II software. Homology modeling of the protein’s three-dimensional structure was conducted using the SWISS-MODEL [39] (https://swissmodel.expasy.org/ accessed on 16 June 2024) automated protein structure prediction server.

### 4.2. Phylogenetic Analysis

The multiple sequence alignment of cassava, *Arabidopsis*, and *Oryza sativa* Shaker proteins was performed using the ClustalW program in MEGA 12 [40] with default parameters. Based on the alignment results, a phylogenetic tree was constructed using the maximum likelihood method with 1000 bootstrap replicates, while other parameters remained at default settings. The phylogenetic tree was refined and visually enhanced using the iTOL website [41] (https://itol.embl.de/ acccessed on 27 June 2025).

### 4.3. Gene Collinearity and Ka/Ks Analysis

The genome files and annotation files of *Arabidopsis*, *Oryza sativa*, and cassava were downloaded from the JGI database. Gene collinearity analysis was performed using MCScanX [42]. Gene duplication events involving duplicated gene pairs were identified using DupGen-finder [43], and visualizations were generated using TBtools-II software [35]. The calculations for Ka, Ks, and Ka/Ks ratios were conducted using the Nei-Gojobori model embedded in the TBtools-II software [35].

### 4.4. Gene Structure and Conserved Motif Analysis

The conserved motif analysis of MeShaker proteins was conducted using the MEME suite [44] (https://meme-suite.org/meme/tools/meme accessed on 20 June 2024) with default parameters and the number of motifs set to 15. Gene structure analysis was performed using the Gene Structure Display Server 2.0 [45] (https://gsds.gao-lab.org/Gsds_help.php accessed on 20 June 2024). The visualization of these data was carried out using TBtools-II software [35].

### 4.5. Cis-Regulatory Element Analysis in Promoter Regions

A 1500 bp sequence upstream of the translation start site of each *Shaker* gene was extracted from the cassava genome to serve as the promoter region. All cassava *Shaker* promoter sequences were submitted to the plantCARE database [46] (https://bioinformatics.psb.ugent.be/webtools/plantcare/html/ accessed on 20 June 2024) for the prediction of cis-regulatory elements within the promoters.

### 4.6. Construction of Protein Interaction Networks

Protein–protein interaction (PPI) network predictions were performed using the STRING database (https://cn.string-db.org/ accessed on 22 June 2024). The resulting predictions were imported into Cytoscape software for refinement and visualization [47].

### 4.7. Gene Expression Pattern Analysis

RNA-seq raw data were downloaded from the NCBI database (accession number GSE82279) [48]. After filtering, alignment, and quantification, the TMM expression matrix was obtained. The expression profiles of *MeShaker* gene family members across various tissues were extracted from the TMM expression matrix and subsequently visualized using R programming.

### 4.8. Plant Growth and Treatment

Cassava germplasms NZ199 and 47-11 were used as experimental materials, representing different levels of potassium stress tolerance, with NZ199 classified as a low-potassium-tolerant germplasm and 47-11 as a potassium-sensitive germplasm, and all experiments were conducted at Hainan University (longitude: 110.326842, latitude: 20.056716). Stem cuttings of 3–4 cm in length from the NZ199 variety were cultivated in pots filled with clean sand. The plants were grown in a greenhouse under controlled conditions (30 °C during the day and 25 °C at night, with a 12 h light/12 h dark photoperiod). Uniformly growing and healthy potted seedlings were selected for long-term potassium stress treatment. Potassium stress was applied using Hoagland nutrient solution with a potassium ion concentration of 0.01 mmol/L for a duration of one month. After the treatment, 3–5 leaves from the top were collected, rapidly frozen in liquid nitrogen, and stored at −80 °C for further use.

### 4.9. Analysis of K^+^ Concentration in Plant Samples

Plant samples were initially dried in a forced-air oven at 80–90 °C for 15–30 min and then further dried at 60–70 °C until they were completely dehydrated. The samples were ground, passed through a 40-mesh sieve, and homogenized. A 0.1–0.5 g portion of the dry sample (accurate to 0.0001 g) was digested with 5 mL of concentrated sulfuric acid and two 2 mL additions of hydrogen peroxide. After the initial vigorous reaction subsided, the mixture was heated until white fumes appeared and the solution turned brown. Following cooling, additional drops of hydrogen peroxide were added incrementally, and digestion continued until the solution became clear or colorless. A final 5 min heating step was used to eliminate excess peroxide. After cooling, the digest was transferred to a 100 mL volumetric flask, diluted to volume with distilled water, and clarified by filtration or standing to yield solution A for potassium determination. The potassium content was measured using flame atomic absorption spectrophotometry (Shimadzu AA-7800 atomic absorption spectrophotometer was sourced from Shimadzu Corporation, located in Kyoto, Japan). Standard potassium solutions of 0, 5, 10, 20, 30, 50, and 70 mg/L were prepared, and their absorbance values were used to construct a standard calibration curve. Three biological replicates were conducted.

### 4.10. RNA Extraction and RT–qPCR

Total RNA was extracted using the Plant Total RNA Extraction Kit (TIANGEN Biotech Co., Ltd., Code: DP432, Beijing, China). The reverse transcription of 1 µg of RNA into cDNA was performed using the Evo M-MLV RT Premix Kit Ver.2. The expression levels of target genes were measured using a Bio-Rad CFX96 real-time PCR instrument and the 2X SYBR Green Pro Taq HS Premix II Kit (Accurate Biology, Ltd., Wuhan, China; AG11702), with four biological replicates for each sample. The PCR cycling program consisted of an initial denaturation at 95 °C for 30 s, followed by 40 cycles of denaturation at 95 °C for 10 s and annealing/extension at 60 °C for 30 s. Melt curve analysis and cycle threshold (Ct) values were determined using CFX software (Bio-Rad). The cassava actin (act) gene was used as the reference for relative expression analysis. Primers for *MeShaker* genes were designed across exons within the CDS sequence and validated for specificity using Primer-BLAST [49]. Primer sequences are provided in Table S2. Gene expression levels were calculated using the 2^−ΔΔCt^ method [24].

## 5. Conclusions

In this study, 13 members of the cassava *Shaker* gene family were identified and classified into five subfamilies. Homology analysis, gene structure and motif analysis, promoter analysis, and protein interaction network predictions were conducted, allowing for a more comprehensive characterization of the family members’ features. Additionally, qRT-PCR was used to analyze the expression profiles of these 13 genes under low-potassium stress. The results showed that all genes were induced by low-potassium stress in leaves. Among them, *MeAKT1.2*, *MeAKT2.1*, and *MeAKT2.2* exhibited opposite expression patterns between roots and leaves, and tissue-specific expression analysis revealed that these three genes were expressed in all tissues. More importantly, these three genes exhibit distinct expression patterns between the potassium-tolerant germplasm and the potassium-sensitive germplasm, with higher expression levels in the leaves of the low-potassium-tolerant germplasm (NZ199) compared to the potassium-sensitive germplasm (47-11). Based on these findings, *MeAKT1.2*, *MeAKT2.2*, and *MeKAT1* were identified as candidate genes involved in cassava’s response to low-potassium stress.

## Figures and Tables

**Table 1:** Information on *Shaker* k^+^ channel gene family members in cassava.

Gene ID	Gene Name	Start	End	Chr	Length (aa)	PI	MV (KDa)	Subcellular Localization	Number of TM Segments
Manes.01G120800	MeSKOR.2	31,695,434	31,712,009	Chr1	828	6.09	94.93	plas	6
Manes.02G078700	MeSKOR.1	6,084,693	6,096,551	Chr2	836	5.98	96.44	plas	6
Manes.03G017700	MeAKT6	1,483,766	1,495,668	Chr3	894	6.43	101.02	plas	4
Manes.03G064216	MeAKT3.2	7,669,159	7,682,497	Chr3	632	7.03	72.63	chlo	6
Manes.06G002600	MeGORK	623,796	637,387	Chr6	830	6.06	94.44	plas	6
Manes.06G050400	MeAKT1.2	17,445,155	17,451,647	Chr6	878	8.06	99.19	chlo	6
Manes.07G018900	MeAKT2.2	2,148,194	2,155,082	Chr7	824	7.28	93.97	plas	6
Manes.10G122000	MeAKT2.1	29,029,863	29,038,057	Chr10	841	6.24	95.91	plas	6
Manes.14G128600	MeAKT1.1	10,233,888	10,239,538	Chr14	877	8.43	99.02	chlo	4
Manes.15G120900	MeKAT2	9,587,853	9,594,437	Chr15	791	6.61	89.75	chlo	6
Manes.16G069300	MeKAT3.1	26,492,008	26,500,663	Chr16	630	6.76	72.34	chlo	6
Manes.16G118800	MeAKT5	32,244,340	32,249,876	Chr16	902	5.98	101.91	plas	6
Manes.17G069300	MeKAT1	26,926,476	26,932,447	Chr17	789	8.47	90.3	chlo	6

## Data Availability

The data supporting the findings of this study are available in the Supplementary Materials of this article.

## Supplementary Materials

The following supporting information can be downloaded at: https://www.mdpi.com/article/10.3390/plants14142213/s1, Table S1. Ka, Ks, and Ka/Ks of *MeShaker* K^+^ channel gene family. Table S2. qPCR primer list. Figure S1. Predicted three-dimensional structure of cassava MeShaker K+ channel protein. The model represents the spatial conformation of the MeShaker protein, highlighting key structural features such as the transmembrane helices, voltage-sensing domain, and pore region responsible for potassium ion transport. Different colors indicate distinct secondary structural elements, in-cluding α-helices and β-sheets. Figure S2. Multiple sequence alignment of Shaker K^+^ channel gene family members from *Manihot esculenta*, *Oryza sativa*, and *Arabidopsis thaliana*. Conserved motifs, including the signature GYGD/GYGE sequences located in the pore region, are indicated with red boxes. The original amino acid sequences of the 13 cassava Shaker members.
