# Supplementary material for: Genome-Wide Identification and Expression Analysis of the Shaker K+ Channel Gene Family in Cassava (Manihot esculenta Crantz) Under Potassium Stress
Source: Plants (Basel). 2025 Jul 17;14(14):2213. doi: 10.3390/plants14142213 (PMC12299194; doi:10.3390/plants14142213)
Supplement: Supplementary file 1 [file plants-14-02213-s001.zip › plants-3709882-supplementary.pdf]

The original amino acid sequences of the 13 cassava Shaker members

>Manes.14G128600
MDTLRNRGVFRVSVCGQEEIEQLSRDGSHYSLSTAVLPSLGA
RSNRRVKLRRFIVSPYDRRYRIWETYLVVLVIYTAWVSPFEFG
FLKKPEGPLSIADNIVNGFFAVDIFLTFFVAYLDKTTYLLVDN
PKKIAWKYTSSWFAFDFISTIPSELAQKISPKPFRTYGLFNMLR
LWRLRRVSSLFSRLEKDRNYNYFWVRCAKLICVTLFAVHSAG
CFYYLIAARYRNPDKTWIGASLGDNFLEKSVWIRYVTSIYWSI
TTLTTVGYGDLHPVNTREMIFDIFYMLFNLGLTAYLIGNMTN
LVVHGTSRTRRFRDTIQAASSFAQRNQLPLRLQDQMLAHLC
LKFRTDSEGLQQQETLDSLPKAIRSSISHYLFYSLLDRVYLFRG
VSNDLLFQLVSEMKAEYFPPKEDVILQNEAPTDLYILVTGAA
DLLVYKNGAEQLVGKVNSGDLCGEIGVLCYRPQLFTVRTKR
LSQLLRLNRTTFLNIVQANVGDGTVIMNNLLQHLKEQKDPI
MEGVLLETENMLAHGRMDLPLSLCFAALRGDDSLLHQLLK
RSLDPNESDNNGRSALHIAASKGSENCVRLLLDYGADPNCK
DSDGNVPLWEAMLGGHEAVARLLIENGASINAGDLGHFAC
TAAEQNSLNLLKEIVRYGGDVTCSRKSGTTALHVAVCEDNT
EIVRFLLDQGADTDKPDNHGWTPRDLADQQGHEEIKFIFRT
CKEPKPVPSVAIPDKKDPGIRYLGRFTSEPAIRPVSGDGSFSVA
EGSWSQNRPRRRTNNFHNSLFGMMSAAHKGEKELPFPVSH
LVVADNYGAKPARVVISCPEKVEIAGKLVLLPKSLEELLEIGA
KKFGLTNAKVLSKNRAEIDDIEVIRDGDHILIVSDRIDH*

>Manes.16G069300
MSETRSPLPLLFRRRSSSEMMKNLASVSSSLLPAFGTVVDEGY
LHLRKYVIAPYDRRYRWWQTFLVALVIYSAWASPFELAFKK
AATDAFMPVDLVVDAFFAMDILLTFFVAYLDKSTYLLVDDH
KKIALRYVTSLWFPMDVASTLPFQFLYRIFTGKMSHGEIFGFL
NLLRLWRLRRVSELFKRLEKDTRFGYFWTRLFKLISVTLFAVH
SAGCFYFWLAAHHKTPENTWIGVEVSDFKHRSIWLCYTYSIY
WSIVTLTTVGYGDLHAVNTGEKIFNMLYMLFNIGLTAYIIGN
MTNLIVHSAVRTFAMRDAINEILRFGSKNRLPEGLKGQMLA
HMQLKFKTAELQQEEVLQDLPKAIRSSIAQHLFHSTVGNTYL
VKGVSEDLISQLVSEMKAEYYPPKVEIILQNEIPTDFYILVSGE
VDVLTYKNGTEQSLSKLGAADMAGEIGVIFNIPQPFTVRTKR
LSQVIRMSHHHFKQMVQPQSEDGKIIISNFIQYLKGLKQEMQ
EEIPFLMELLDDMNATHTETNEELQNPGALNSHAETNTEGR
PETSSPISRKCPIRVIIHGHHSNESPKEGDISGKLIHLPDSIEDLF
RLAEKKF
GKQGNTILMEDGSLVEDLVALRENDHLFIF*

>Manes.03G017700
MKKKSKRLLSWGQVWPDPEERDMEDRGDEEIEKFSKDDRSY
FSLTGEILPSLGKNAVSNRRIELRRFIISPFDPRYRTWDTFLVFL
VFYTAWASPFEFGFLERPTRALAVTDNVVNAFFTVDIVLTFF
VAYVDKSTYNLVVNRKKIALKYAKTWLAFDVISTIPSELVRSI
LPSKLQSYGYFSMLRLWRLRRVSLFFARLEKDKNYSYFWVRC
LKLICVTLFVVNIAGCFYYRLAFTYHDQANTWIGSVWEHYQ

TQSMWVRYVTSLYWSITTLTTTGYGDLHAVNEREMIFVMCY
MMFDLGLTAYLIGNMTNLVVHATSRTRQFRDTIQAASNFA
QRNQLPVRLQDQMVAHLSLKYRTNSEGLHQQETIDSLPKAI
RSSIANYLFYNLVNEVYLFRGVSNDLLFQLVAEMKPEYFPPK
EDVILQNEAPTDMYILITGAVELVVKTGGVEKTVGEARAGD
VVGEIGLLCYKPQMFTARTKRLSQLLRMNRTAFLNIVQSSVG
DGTIIMNNLLQHLKELDNPVMQAILAETERMMARGSLDLPL
TLCFAAMRGDDLLLHQLLKRGADPNELDENGRTAMHIAAS
NGSEHCVILLLEYGADPNKKDSEGNVALWDALLGKHESVIK
LLVENGATMSSGDVGQFALTAIEQNNLDLLKDIVNYGGNVT
LPTSNGTIALHTAISEGNTEIVKFLLDQGSDVDMPDVHGWT
ARGLADHQGHEEIQALVQTMQKKEKKTVHTVRIKQQGKLC
LEKPIARYSSEPAIPPCSKQDVLPQTPTMLLPETRQRRRADTY
QNSLLGIMSAANTGESEIITSPTGTAGITSFSNQARVTISCPEK
GEVNGKLILLPKSLEELLDIGAKKFGIIPTRILTKEGAEIEELELI
RDGDHLVLVSNADTRT*
>Manes.06G002600
MMMMMQRKQERRDSDSEEEEFVEEKSDNKPQWKKLLCLFR
NDSIGGIGGEIVRDGNGSSVAGGSSSASHRGFIIRPDDWWYT
AWTHFILIWAIYSSFFTPLEFGFFRGLPENLFLLDIAGQIAFLID
IIVRFFVAYRDTHSHRLVYNRNLIAIRYLKSRFLVDILGCLPW
DAIYKACGRKEAARYMLWIRLSRARRVSEFFERLEKDIRINYL
FTRIVKLLVVELYCTHTAACIFYYLATTLPPSEEGYTWIGSLQ
MGDYHYSHFRDIDLWKRYITSLYFAIVTMATVGYGEIHAVN
VREMIFVMVYVSFDMILGAYLLGNMTALIVKGSKTEKFRDK
MAELIKYMNRNNLGKGISNEIKGHLRLQFDRSYTEAAVLQDI
PSSLRAKISQKLYEPYIKEVHLFKGCSLGFIKQIAIKVHEEFFLP
GEVIIEQGQIVDQLYFVCHGELVKEELGKEGNDETEEPMCLQ
AYSSFGEISFLCNTPQPHTVRVRELCRVLRLDKRSFTEILEIYFS
DGRTILNNLLEGKDSNLQNELLESDVTLHIEKSESVLATRLN
CAAYDGDFYRLKRLIGAGADPNKTDYDGRSPLHVAATKGH
EDITLFLIEQGVDVNISDKFGNTPLLESVKGGHDEVGSALVR
AGATLEIDDAGGFLCMAVARRDLGLLKRALANGMNPNAK
NFDCRTPLHVAASEGLYPMAKLLLEAGASVFSKDRWGNTPL
DEARLGGNKNFINLFETARTSQISELSDCHGGIQAVAEKQRR
KCTVYPFHPWDPTEKRREGVVLWVPETMEEIVKAAMEQLN
CTSSCILSENGGKIIDVNMINDNQKLFLVSES*
>Manes.15G120900
MSLSCAKNFFQRFCSEDVQMEGIPPGNFFSSDLLPSLGARINQ
ATILRRYIISPYSSRYRAWEMWLVVLVIYSAWISPFELAFLTYK
KDDALFIIDNIVNGFFAIDIVLTFFVAYLDSHTHLLVDDPKKI
AIRYISTWFLFDVCSTAPFQSISLLFTNQSSSEIGFRLLNMLRL
WRLRRVSSLFARLEKDIRFNYFWTRCTKLVSVTLFAVHCAGC
FNYLIADRYPDPKRTWIGAVNPNFKEDSLWNRYVTAMYWSI
TTLTTTGYGDLHAENPREMLFDIFYMLFNLGLTAYLIGNMT
NLVVHWTSRTRNFRDTIRAASEFATRNQLPPHIQDQMLSHL
CLKFKTEGLKQQKTLNGLPKAIRSSIAYHLFYPIVEKVYLFQG
VSHDFLFQLASGMEAEYFPPKEDIILQSEAATDLYILVSGTVD
LMCYVDGIEQVIGKANAGDIFGEIGVLYNRPQPFTARTSELS

QILRLTRTSLINAIQANTADGCIIMNNLFKKLQGLESSRTGFD
YQNKDPGTILGEWCDGVPKEGCSSEAGCQNNLHGDRLFHE
VGDSSAEESAAKGKSKRGTGHNFIPHVGDVNSTIEDDQNSL
VTAVRNGHIEMVKLLLEEGANADKPDATGSTPKALAAQQG
HKNIYDLLLSYENRRKLIEHKIDLVETESEEAKNNQGQHKGV
GGPNCFYFHSKMVPTNSSSRPYSCLNNKEPKNLTKKRVTIH
MQFHNRSTLKRPPGKLITLPDSIEELLRIGGEKFGGYKFTRVIN
ADNAEIDDISVIRDGDHLYLLQNDTEILDYNVT*
>Manes.17G069300
MSLSYAKNFFHRFCSDDIQMEGIPHGSFFSSDLLPSLGARINQ
ATTLRRHIISPYNSRYRAWEMWLVVLVIYSAWISPFEFAFLTY
KKDDALSIIDNIINGFFAIDIVLTFFVAYLDSHTYLLVDNPKKI
AIRYISTWFLFDVCSTAPFQSLSLLLTNQSSSQIGFRLLNMLRL
WRLRRVSSLFARLEKDIRFNYFWTRCTKLISVTLFAVHCAGCF
NYLIADRYPDPKRTWIGAVNPYFKEDSLWDRYIAAIYWSITT
LTTTGYGDLHAENPREMLFDIFYMLFNLGLTAYLIGNMTNL
VVHWTSRTRNFRDTIRAATEFATRNQLPPHIQDQLLSHLCLK
FKTEGLKQQETLNGLPKAIRSSIAHHLFYPIVEKVYLFQGVSY
DFLFQLVSEMEAEYFPPKEDIILQSEASTELYILVSGTVDLMSC
ADGIYQVIGKATAGDIFGEIGVLYCRPQPFTARTSEISQILRLS
RTSLINTIQANMEDGHIIMNNLFKKLQGSESTGFDYQNKAPG
KILNAWCDEGSNEGWGSEAICQNYSQEEPMMQEAGNSSAE
ELEARGKSRGIGHNFLTQGPDIHSTIEDDQMALQFHTAVHK
GHIEMVKILLEGGANAEKSDAIGRTPKALAEQQETKSIYDLL
NNKNTRKIDEYIIDFIEPKSDETKKPSKQKGVGGPNYFNVHS
KMVPTNSSPRPHSCPNDGETKNTKKRVTIHMQFHNRSKLQR
PFGKLIMLPDSIEELLRIGGKLCMTSNEHLNVNLNIRNKNYIL
ISSVNRSKNKCKQKNEQRHQTLHKN*
>Manes.07G018900
MEMKSSWENHHEEKKQSNHYEEDDTSLSLSSLSKIILPPLGVS
SYNHNPIETKGWIISPMNSKYRCWETYMVVLVAYSAWVSPF
EVAFLKSNPNKGLYVADSVVDLFFAIDIVLTFFVAYIDSTTHL
MVRDRRKISIRYLSTWFSMDVASTIPFEALGYLFTGKRKMGLS
YSLLGMLRFWRLRRVKQLFTRLEKDIRFSYFWVRCTRLLFVTL
LLVHCAGCLCYLLADRYPHQGRTWLGSVNPNFRETSLRNRY
ISALYWSVTTMTTVGYGDLHAVNTGEMIFIIFYMLFNLGLTA
YLIGNMTNLVVEGTRRTMEFRNSIEAASNFVCRNRLPPRLKE
QILAYMCLRFKAESLNQNHLIEQLPKSICKCICQHLFLPIAEK
VYLFKGVSREILLLLVAEMKAEYIPPREDVIMQNEAPDDVYII
VSGEVEIIDSALEKERIFGILQSGDMFGEVGALCCKPQSFTFRT
KTLSQLLKLKTSALIETMQIKQEDYVAIIKNFLQHHKKLKDFK
IGEFIAEGGEEDGDPNMAFNLLTAASAGNAAFLEELLRAKL
DPDIGDSKGRTPLHFAASKGHEDCALALLRHGCNIHLKDVN
GNTALWEALSSKHQSVFRILYHFANVSDPHTAGDLLCTAAK
RNDLTMMNSLLKHGLNVDSKDRQGKTAVQIAMAQNYIDM
VDLLVMNGADVSAANSSEFCSTTLNKMLQRRESGHRITMPD
TVTSDEVILKMDQEEKQCKSSEKSNELKYTRVSIYRGHPLVRK
ETCCRQAGRLIRLPNSMEELKSIAGEKFRFDARNAMVTDEEG
SEIDSIEVIRDNDKLFIVEDPTPFM*

>Manes.06G050400
MDNLRNRGVFRVSVCGQEELEQLSRDGSHYSLSTAVLPSLG
ARSNRRVKLRRFIVSPYDRRYRIWETFLVVLVIYTAWVSPFEF
GFLKKPEGPLSIADNVVNGFFAIDIVLTFFVAYLDKTTYLLVD
DPKKIAWKYTRSWFVFDFISTIPSELARKISPKPFQSYGLFNML
RLWRLRRVSALFSRLEKDRNYNYFWVRCAKLICVTLFAVHS
AGCFYYLIAARYPNPEKTWIGASLGDNFLQQSVWIRYVTSMY
WSITTLTTVGYGDLHPVNTREMIFDIFYMLFNLGLTAYLIGN
MTNLVVHGTSRTRRFRDAIQAASSFAQRNQLPLRLQDQMLA
HLCLKFRTDSEGLQQQETLDSLPKAIRSSISHYLFYSLLDRVYL
FRGVSNDLLFQLVSEMKAEYFPPKEDVILQNEAPTDFYILVTG
AVDLLVYRSGGEQVVGQATSGELCGEIGVLCYRPQLFTVRTK
RLSQLLRLNRTTFLNIVQANVGDGTIIMNNLLQHLKEQKDPL
MEGVLHETENMLARGRMDLPLSLCFAALRGDDSLLHQLLK
RGLDPNESDNNGRSALHIAASKGSENCVLLLLDYGADPNCK
DSDGNVPVWEAMLGGHEAVAKLLIENGASINFGDVGHFAC
TAAEQNSLNLLKEIVRYGGDVTSPRKSGTTALHVAVCEDNT
EIVRFLLEQGADIDKPDIHGWTPRDLADQQGHEEIKFIFQTC
KEPKAQPVVTIPENNEPGIRYLGRFTSEPTIRPVSRDGSFSATD
GSWSQSRPRRRTNNYHNSLFGMMSAAHKGEKESPFPVGHII
AADNYGANPPRVIISCPEKVEIKGKLVLLPKSLRELAEIGAKK
FGLTNAKVLSKERAEIDDIEVIRDGDHILIASDQTKET*
>Manes.02G078700
MGGHREREIMNEEEEEKDEKQEDREYEVTGVRDRIHSSLGSR
FNLIENEFGIESGNRRMFSRESVINGIRYVSRGLFIHPENRWYR
AWTKFILIWAIYSSFFTPMEFGFFRGLPENLFILDIVGQLAFLF
DIVLQFFIAYRDSQTYRMIYKRTPIAIRYLKSHFFIDLLACMPW
DMIYKACGRKEEVRYLLWIRLSRVRKLTNFFQNMEKDIRINY
LFTRIVKLTAVELYCTHTAACIFYYLATTLPSSEEGYTWIGSLK
MGDYSYSHFREIDIWKRYVTSLYFAIVTMATVGYGDIHAVN
MREMIFVMIYVSFDMVLGAYLIGNMTALIVKGSKTEKFRDK
MTDLIKYLNRNRLGRDIRNQIKGHLRLQYESSYTEASVLQDIP
ISIRAKISQTLYMPYIEKVPLFKGCSAEFINQIVIRLHEEFFLPGE
VIMEQGNVVDQLYFVCHGVLEVVGTGEDGSEETVSLLQPNS
SFGEISLVCNIPLPYTIRVCELCRLLRLDKQSFSNILEIYFYDGR
KILNNILEGKESSLRDKQLESDLAFHIGKQEAELALRVNSAAY
HGDLYELKGFARAGAGLNRTDYDGRSPLHLAASRGYEDITR
FLIQEGVDINIKDKFGNTPLLEAIKNGHDHVASLLVEEKAELS
LDDAGSFLCSVVSHGDSDLLKRILSNGIDPNSKDYDHRTPLH
VAASEGLHMMAKLLIEAGASVFSKDRWGKTPLDEGRMSGN
KNLIKLLEDAKSAHLSEFPPHSQEITVTEKIQRRKCTIFPFHPW
GPKEEKRSGIVLWVPQTLEELIKTAVEQLQLPQDCYMLTEDA
GKILDVDMIDDGEKLYLISETHKI*
>Manes.03G064216
MSETRAPLPWLFRRRSSTEMMKNLASVSSSLLPAFGTVVDEG
YLQLRKRVIAPYDRRYRWWQTFLVALVIYCAWASPFELAFK
KAATGGFMAVDLVVDVFFAIDILLTFFVAYLDKSTYLLVDDH
KKIALRYVTSLWFPMDVASTLPFQLVYRIFTGKTSESEVFRVL
NLLRLWRLRRVSELFERLEKDIRFSYFLTRLVKLISVTLFAVHS

AGCFYFWLASHHKNPDNTWIGIKIQDFKHRSIWLGYTYSIY
WSIVTLTTVGYGDLHAVNTGEKIFNMFYMLFNIGLTAYIIGN
MTNLIVHAAVRTFAMRDTINEVLRYASKNRLPEGLKGQML
AHMQLKFKTAELQQEEVLEDLPKAIRSSIAQHLFRSTVENTY
LFKGVSEDLVIQLVSEMKAEYYPPKVEIILENEIPTDFYIMVSG
ELDVLTYKNGTQQVLSKLGAADVAGEIGVIFNIPQPFTMRTK
RLSQVIRMSHHHFKLMVQPESEDGKTIISNFIQYLKGLKQEM
QKEIPFLMELLRDMNVEHNATNEGLQNPAALNSHGDTSDE
ERPETSSPISSKNPIRVVIHGHHPNESPAEGQGDTSGKLIHMP
DSIEDLFRLAEKKFGKRGNTILMEDGSQVEELVALRENDNLFI
F*
>Manes.01G120800
MGGDRERDIRNRKKEDEQKESEYEIEDVSDAIDSYRGSGFNH
IENELGLEAGARRKFSRDSVINGIRYVSRGLFIHPESRWYRAW
TKFILIWAIYSSFFTPLEFGFFRGLPENLSILDIVGQIAFLFDIILQ
FFIAYRDSQTYCMVYKRTPIALRYLKSHFFIDLLGCLPWDIIYK
ACGRKEEVRYLLWIRLSRVRKVTDFFQKMEKDIRINYLFTRII
KLTAVELYCTHTAACTFYYLATTLPSSEEGYTWIGSLKMGDY
SYSHFREIDIWKRYVTSLYFAVVTMATVGYGDIHAVNLREMI
FVMVYVSFDMVLGAYLIGNMTALIVKGSKTEKFRDKMKDLI
NYMNKNRLGKDIRNQIKGHFRLQYESSYTEASVLQDIPISIRA
KISQTLYMPYIENVPLFKGCSGEFINQIVIRLHEEFFLPGEVIME
HGNVVDQLYFVCHGVLEVVGSGGDGSEETVSLLQPNSSFGEI
SILCNIPQPYTIRVCELSRLLRLDKQSFSNILEIYFYDGRKILNNI
LEGKESSLRDKQLESDIAFHIGKQEAELALRVNSAAYHGDLY
ELKSFVRAGADPNRTDYDGRSPLHLAASRGYEDITLFLIQERV
DINIKDKFGNTPLLEAVKYGHDQVASLLVKEGADLSVDDAG
SFLCLIVSKGDSDLLKRILSNGIDPNSKDYDHRTPLHVAASEG
IYIMAKLLLEAGASVFSKDRWGKTPLDEGRMSGNKNLIKLLE
DAKSAQLSEFSCNSEITDKIHRRKCTIFPFHPWGPKEHKRPGI
VLWVPETMEELIKTAAEQLQLPYDSCILTEDAGKILDTNMID
DGGKLYLVSETR*
>Manes.16G118800
MFMKRSRHWLYFGQGLTEEEEERGMGGHDDDKIEKFSVDD
RSYFSLTDYILPSLGKNAVSNRRMELRRFIISPFDPRYRTWDTF
LVFLVFYTSWASPFEFGFLEWPVGALAIVDNVVNAFFAVDIV
LTFFVAYLDKYTFLLVDNRKKIAFRYAKTWLVFDVVSTFPSEL
LRSIFPDRLQSYGYLSMLRLWRLRRVSRFFARLEKDRNFSYFW
VRCTKLIFVTLFVINMAGCFFYRLAITYYDPTKTWIGSVWNDF
EQHSLSTRYVTSLYWSITTLTTTGYGDLHAVNEREMIFTMFY
MMFDLGLTSYLIGNMTNLVVHATSRTRKFRDTIQAASSFAQ
RNQLPVRLQDQMLAHLSLKYRTDSEGLHQQETIDSLPKAIRS
SIANYLFYNFVNEVYLFKGVSNDLLFQLVAEMKAEYFPPKED
VILQNEAPTDMYILITGAVELIVPRGPTEQVVGEAKTGDVVG
EIGLLCYRPQMFTVRTKRLSQLLRLNRTAFLNIVQASVGDGTI
IMNNLLQHLKELNDPMMEGILAETERMLAHGRMDLPLNLC
FAAMRGDDLLLHQLLKRGLDPNDLDENGRTALHIAASNGS
EHCVVLLLEYGADPNKKDSEGNVPLWEALLGKHESVVKLL
VDNGATISSGDVGQFTLAAIEQNNLDLLKEIVNYGGDVTLLT

SSGNIPFHSAISGGNTEIVQFLLDQGADVDRPDVHGLTARGL
ADHQGQEEIQALIRTRQETEKKQVPTMPLQQQQGKLHLWK
TIAKCGNEPSTPRPSTPLPSNKDVMPASPGVILTDNRQRRKVS
PFHNSLLGIMSAANAGDHEMISSPSGAAAAGAGFTSLSYPAR
VTISCPEKGEVEGKLILLPKSIQELLDIGAKKFGFFPTRILTKEG
AEIEDLELIRDGDHLVLASNG*
>Manes.10G122000
MEMRSTPSNDLYHLPFTMKRSWRNHHGHPQTPHHHHHQE
DDTSLSVSSLSKIILPPLGVSSYNHNPVETKGWIVSPMNSKYR
CWETFMVVLVAYSAWVYPFEVAFLNSSPNKMLYITDNIVDL
FFAIDIVLTFFVAYIDSRTQLLVRDRTKISIRYLSTWFLMDVAST
IPFEALAYFFTGKHSMGLSYSLLGMLRFWRLRRVKQLFTRLE
KDIRFSYFWIRCARLIIVTLFLVHCAGCLYYLLADRYPHQGRT
WIGAVIPNFRETSLWIRYISALYWSITTMTTVGYGDLHAVNT
MEMIFIIFYMLFNLGLTAYLIGNMTNLVVEGTRRTMEFRNSIE
AASNFVCRNRLPPRLKEQILAYMCLRFKAESLNQNHLIEQLP
KSICKSICHHLFLPTVEKVYLFSGVSREILLLLVAEMKAEYIPP
REDVIMQNEAPDDVYIIVSGEVEIIDSDLEKELVVGTLQSGDM
FGEVGALCCKVQSFTFRTKTLSQLLKLKTSTLIDTMQTKQED
YVAIIKNFLQHHKKLKGLKLGESLVDDGEEDGDPNMAFNLL
TVASTGNAAFLEELLRAKLDPDIGDSKGRTPLHVAASKGHE
DCVLALLRHGCNINLRDVNGNTALWEALSSKHQSVFRILYH
FSNIDDPHTAGELLCKAAKENDLTMMKELLKHGLNVDAKD
RQGKTAVQIAMAQNYVDMVDLLVMNGADVSAANTSEFSS
TTLNEMLQKREIGHRITVPDTVTSDEVILKRNQEEEEGNSSGK
SNGWECRRVSIYRGHPLIRKETCCLEPGRLIRLPNSMEELKSIA
GEKFGFDARNAMVTDEEGSEIDSIEVIRDNDKLFIVEDPNSSM
*

Supplementary A

Supplementary S1

Table S1. Ka, Ks, and Ka/Ks of *MeShaker* K^+ channel gene family.

Seq1	Seq2	Ka	Ks	Ka/Ks
MeSKOR.2	*MeSKOR.1*	0.0631	0.3703	0.17
MeSKOR.2	*MeGORK*	0.2314	1.4423	0.16
MeAKT6	*MeAKT1.1*	0.2523	1.4397	0.18
MeAKT6	*MeAKT5*	0.1175	0.3525	0.33
MeKAT3.2	*MeKAT3.1*	0.0678	0.3742	0.18
MeKAT1.2	*MeAKT5*	0.2352	1.4068	0.17
MeAKT2.2	*MeAKT2.1*	0.0753	0.3923	0.19
MeKAT2	*MeKAT1*	0.1076	0.2746	0.39

Table S2. qPCR primer list.

Gene Name	Sense Primer (5′-3′)
MeAKT6-F	TTTCGAGAATGAAGCACCCA
MeAKT6-R	TGACTCAATCGTTTTGTCCGAG
MeGORK-F	TGACAATAAGCGCGAGTGG
MeGORK-R	TGAATAAATCGCCCATATAGT
MeKAT2-F	TTCCCCCTACAGTTCGCCTTA
MeKAT2-R	AGGAGATGAGTATGGTGGTG
MeKAT1-F	GCTGAGAACCCCAGGGAAATGC
MeKAT1-R	GCGGCTGGTCCAGTGAACAAC
MeAKT2.2-F	GTGCTGGGTGCCTGTGCTA
MeAKT2.2-R	GGAGGCTACCATACCAACAG
MeAKT1.2-F	AGACGGTTCATGGAGCCAAAGC
MeAKT1.2-R	CCCCTTGTGAGCAGCAGACATC
MeSKOR.1-F	CTTATCTTATTGGTAATAGACCGC
MeSKOR.1-R	GTCCCTTCCAAGTGTTTCTG
MeAKT3.2-F	TGATGGAATTGCTGCGTGAC
MeAKT3.2-R	TCTGCTGGACTTCATTTGGA
MeSKOR.2-F	GTTGCTGCCTGGGACA
MeSKOR.2-R	TTTGAAAAAAATCAGTATCTTGCG
MeAKT5-F	GGTGAGATTGGTCTGCTGTTA
MeAKT5-R	GCAGTAGCTGACCGCAGT
MeAKT2.1-F	CCCCGAGTTGAAAGAGCAG
MeAKT2.1-R	CATCTCAGACACAGGAGCA
MeAKT1.1-F	GCCAGCTATTCGCCCTGTGTC
MeAKT1.1-R	CCCCTTGTGAGCAGCAGACATC
MeKAT3.1-F	TTTTGGAAGTGTGTGGAGAA
MeKAT3.1-R	GGCATAAAAGGGTGGGTGG
MeAact-F	TGGATTCTGGTGATGGTGTGAGT
MeAact-R	CCGTTCAGCAGTGGTGGTGA

Supplementary B

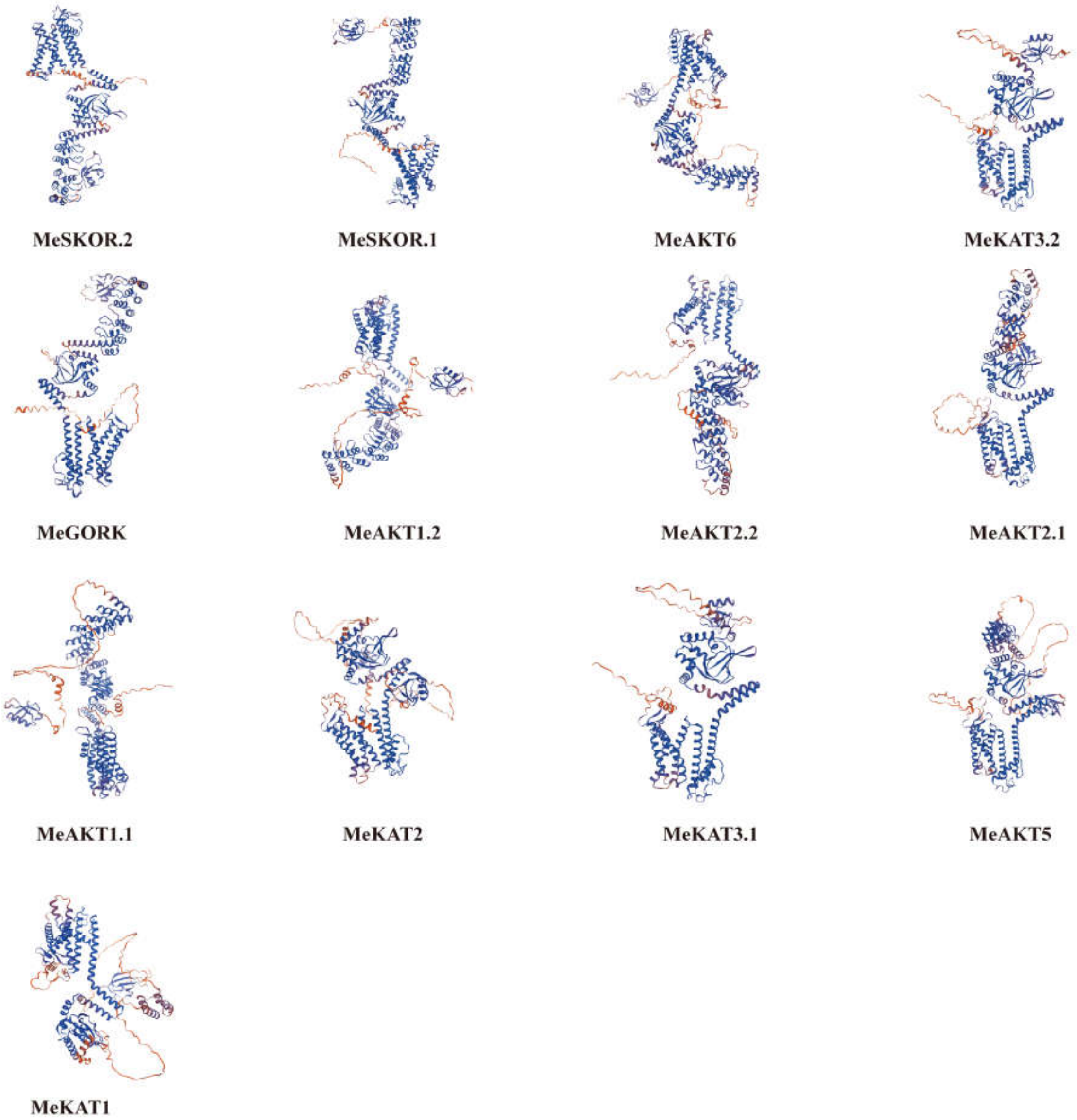

Figure S1. Predicted three-dimensional structure of cassava MeShaker K^+ channel protein. The model represents the spatial conformation of the MeShaker protein, highlighting key structural features such as the transmembrane helices, voltage-sensing domain, and pore region responsible for potassium ion transport. Different colors indicate distinct secondary structural elements, including α-helices and β-sheets.

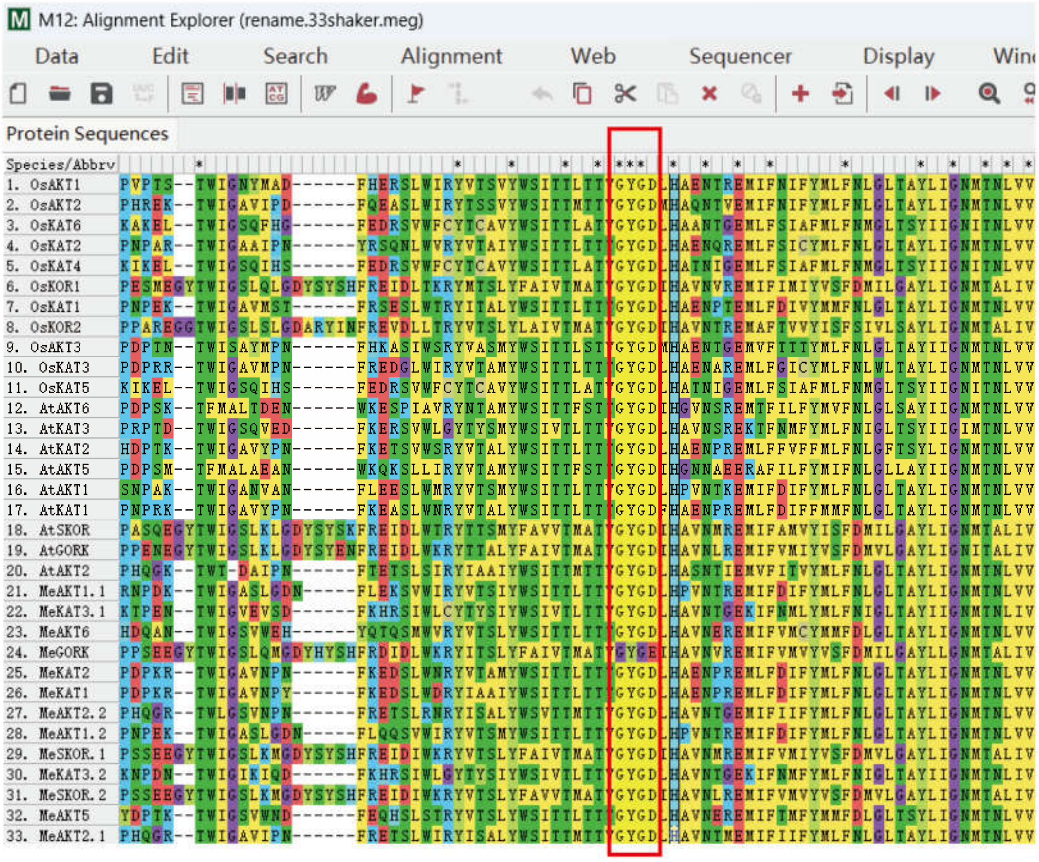

Figure S2. Multiple sequence alignment of Shaker K+ channel gene family members from *Manihot esculenta*, *Oryza sativa*, and *Arabidopsis thaliana*. Conserved motifs, including the signature GYGD/GYGE sequences located in the pore region, are indicated with red boxes.

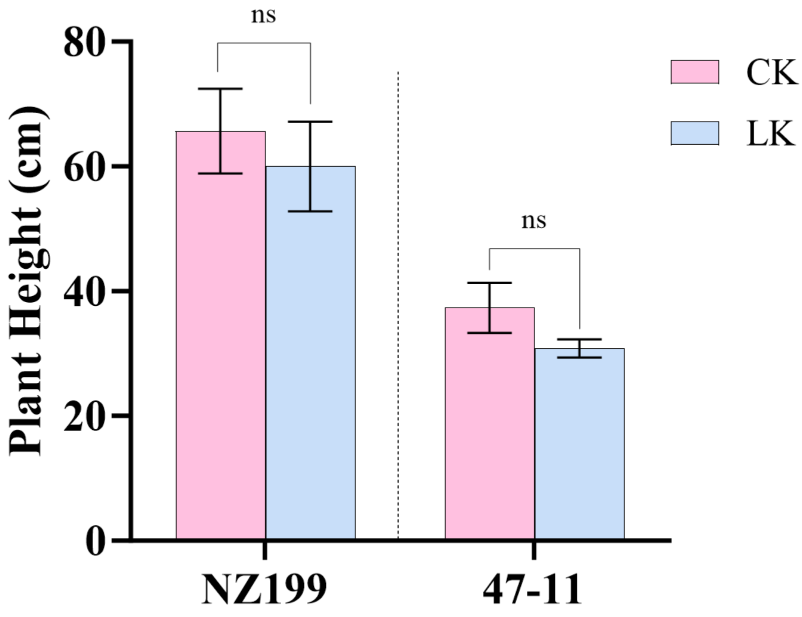

Figure S3. Plant height of NZ199 (low-potassium-tolerant germplasm) and 47-11 (potassium-sensitive germplasm) under potassium treatment,

Statistical significance was determined by one-way ANOVA, where * denotes $p < 0.05$, ** denotes $p < 0.01$, and ns indicates no significant difference. The error bars represent the average variance of three biological replicates.
